# Development of Image-Based Water Level Sensor with High-Resolution and Low-Cost Using Image Processing Algorithm: Application to Outgassing Measurements from Gas-Enriched Polymer

**DOI:** 10.3390/s24237699

**Published:** 2024-12-01

**Authors:** Ji Hun Lee, Jae Kap Jung

**Affiliations:** 1 Hydrogen Energy Group, Korea Research Institute of Standards and Science, Daejeon 34113, Republic of Korea; ljh93@kriss.re.kr; 2 Department of Measurement Science, University of Science and Technology, Daejeon 34113, Republic of Korea

**Keywords:** water level sensor, crescent-shaped water level image, image processing algorithm, performance test, high resolution, low cost

## Abstract

A high-resolution and low-cost image-based water level sensor was developed using an image processing algorithm. The sensor measures water levels in six channels simultaneously. The image processing algorithm automatically identifies water level images and determines the water levels by analyzing the brightness of the images. The measured water levels were verified by comparison with the calibrated water levels using known length standards. The performance test results of the developed water level sensor were compared with those of commercial water level sensors, demonstrating a superior resolution of 0.06 mm and an inexpensive cost of USD 80. In addition, the developed sensor demonstrated an accuracy of 0.9%, a stability of 0.3%, an adjustable measurement range, and an instantaneous response time. In conclusion, the image-based water level sensor that was developed provides a reliable method for real-time visual monitoring of water levels in six channels simultaneously.

## 1. Introduction

Liquid level sensors are devices that measure the volume and height of specific objects of interest, including water, chemicals, slurries, and other fluids. These sensors are used to control the volume of stored liquids and monitor the liquid levels continuously in numerous applications such as water storage management, automated fluid transferring systems, chemical waste monitoring, and medical liquid measurement [1,2,3,4,5,6,7]. In particular, sensors for water level detection are essential for regulating water availability in residential and agricultural water storage tanks [8,9,10,11,12], as well as for monitoring gas flow rates through tubes and gas ejection through gas-permeable membranes [13,14,15,16,17,18].

Water level sensors can be classified according to their detection methods, including float-based, ultrasonic, radar, and capacitance methods. Float-based water level sensors are traditional sensors that measure the height of the float corresponding to the water level in a simple but low-resolution manner. Ultrasonic and radar water level sensors use transducers to send and receive signals containing distance information about the water level with a resolution of 1.0 mm [19,20,21]; however, these detection methods can be affected by vibrations and bubbles on the water surface that cause signal scattering and measurement errors. Furthermore, monitoring signals with high resolution significantly increases the operating cost. Capacitance water level sensors measure the dielectric constant of water using electrodes immersed in water. Water level measurement via capacitance methods is obtained by precalibration between the water level and the capacitance, with good linearity of greater than 99% [22,23]. Although capacitance level sensors exhibit fast response times of less than 1 s and a high resolution of 0.1 mm [24,25], they can induce an inherent risk of electrode contamination due to their direct contact with water, which can lead to a decrease in resolution.

In recent studies [26,27,28,29,30,31,32], image-based water level sensors have been proposed to improve sensor performance in a cost-effective and non-contact manner. These sensors capture images of water levels using a digital camera and automatically determine the water level using image processing algorithms. The performance of image-based water level sensors is highly dependent on various factors, including the image quality, local environmental conditions, and the algorithms used in the image processing [33,34,35,36]. Although high-quality images provide good resolution with the sensor, the resulting increase in data volume inevitably leads to longer processing times. Qiao et al. [35] measured the water level by processing the images of the numerical markings on graduated rulers (staff gauges) partially immersed in the water under various environmental conditions such as bright daylight, infrared lighting at night, rainfall, transparent water, and turbid water. These water level measurements under various environmental conditions exhibited an error of less than 10.0 mm compared with manual readings, except in turbid water, where significant staining of the staff gauge near the water surface resulted in errors exceeding 50.0 mm. This means that the performance of image-based methods can vary depending on the local environmental conditions. Wang et al. [36] combined an algorithm to restore the images obscured by stains with the numerical image processing algorithm, reducing the error to 15.8 mm when using the same image of the turbid water mentioned above. However, errors in other environmental conditions increased. This suggests that the sensor performance can depend on the image processing algorithm employed, even under the same conditions. Therefore, image processing algorithms used in water level sensors are sensitive to various factors and require an optimized system suited to the intended application.

Although many image-based methods for water level sensing have been proposed, only a few studies have systematically evaluated the sensing performance. The performance of the water level sensor should be evaluated in various aspects such as accuracy, sensitivity, resolution, and cost. Furthermore, the individual factors of the sensor performance should be investigated under controlled environmental conditions. Gilmore et al. [37] investigated several sources of uncertainty for an inexpensive image-based water level measurement system in a controlled laboratory environment, including image resolution, lighting effects, lens distortion, and water meniscus. They concluded that evaluating performance under controlled conditions is essential to systematically improve the reliability of the sensor and determine field applicability.

In this work, we propose an image-based water level sensor that uses a low-cost water level sensing system and an image processing algorithm for high-resolution determination of the water level. The sensing system was designed to measure the water level in six channels simultaneously using a digital camera and six glass tubes in a laboratory environment. The image processing algorithm automatically identifies the water level image inside the glass tube, which is observed as a white crescent shape. Then, the center of brightness of the crescent-shaped image is computed as a particular pixel location for precise water level determination. The decrease in the water level is induced by gradual outgassing from gas-enriched specimens under high pressures [38,39,40]. Through the outgassing measurements, the proposed sensor monitors both water levels and gas amounts in six channels in real time.

In the following sections, we describe in detail our image processing method for water level sensing. Section 2 provides the method for fabricating the specimens for a water level decrease and the volumetric method for water level measurement. Section 3 explains the proposed system for water level sensing with the program that we developed. The image processing algorithm for water level determination is presented step by step in Section 4. In Section 5, various aspects of the performance of the water level sensor are evaluated. The results of this performance evaluation are compared with those of commercial water level sensors. We then summarize the characteristics of the developed sensor technology.

## 2. Materials and Methods

### 2.1. Preparation of Gas-Enriched Specimen

The proposed water level sensor detects a decrease in the water level inside a glass tube. To simulate a gradually decreasing water level, we utilized polymer specimens enriched with high-pressure, water-insoluble gas (air, nitrogen: 80%; oxygen: 20%). The water level inside the glass tube was decreased by the water-insoluble gases emitted from the enriched specimen [39,40,41].

**Figure 1:**
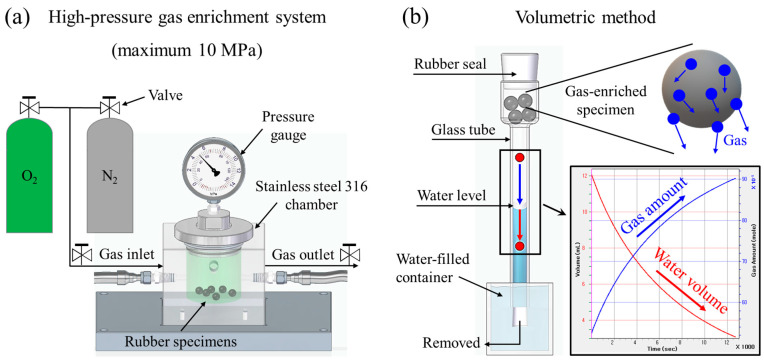
The volumetric method using an ungraduated glass tube after high-pressure enrichment into the specimen. (**a**) System for enriching high-pressure gas in specimens. (**b**) Volumetric method for measuring the decreasing water level versus time elapsed by outgassing from gas-enriched specimens. The blue color in the glass tube represents the water. The gray spheres indicate gas-enriched specimens. The blue spheres represent the emitted gas. The water level is translated into the water volume (indicated by the red curve) and the gas amount (indicated by the blue curve).

As shown in Figure 1a, a chamber (inner diameter: 40 mm; height: 80 mm) fabricated from stainless steel 316 was used for high-pressure gas enrichment at 298 K. Commercially produced spherical rubbers with a diameter of 10.0 mm were used as the polymer specimens for high-pressure gas enrichment. If the specimens occupy a significant portion of the inner volume of the chamber, the inner pressure of the chamber can decrease due to gas enrichment in the specimen. The sizes of the chamber and specimen were determined to ensure stable enrichment without a pressure decrease. We prepared four gas-enriched specimens per channel, as shown in Figure 1b. A total of 24 specimens for six channels occupied only 12.5% of the chamber volume, which enabled stable enrichment. The chamber containing the specimens was purged five times with O_2_ at 1 MPa before gas enrichment. O_2_ was first pressurized at 2 MPa, followed by N_2_ at 8 MPa, injecting a total gas pressure of 10 MPa into the chamber. Since there is no standard to specify the gas pressure, the gas pressure of 10 MPa was arbitrarily determined to sufficiently enrich the gas in the specimen. The specimens were enriched with gas for 24 h. The saturation time is the time required for the specimen to be enriched fully with gas, which is proportional to the square of the diameter of the spherical specimen [42]. The specimen with the diameter of 10.0 mm was fully enriched with gas within 24 h. After 24 h, a valve was opened, and the chamber was subsequently depressurized to atmospheric pressure. The time at which the chamber reached atmospheric pressure (t = 0) was recorded.

### 2.2. Volumetric Method

Figure 1b shows the volumetric method for measuring the decrease in the water level versus the elapsed time [43,44]. The measurement procedure of the volumetric method using a dedicated glass tube was as follows: First, the water was added into the glass tube packed at the bottom with a rubber seal. Then, the gas-enriched specimens were loaded into the empty space at the top of the glass tube, and the tube was sealed. Finally, the bottom rubber seal was removed inside the water-filled container. According to the U-tube manometer principle [45], the water level decreased, indicated by the red vertical arrow in Figure 1b, as the amount of outgassing from specimens increased (blue vertical arrow).

**Figure 2:**
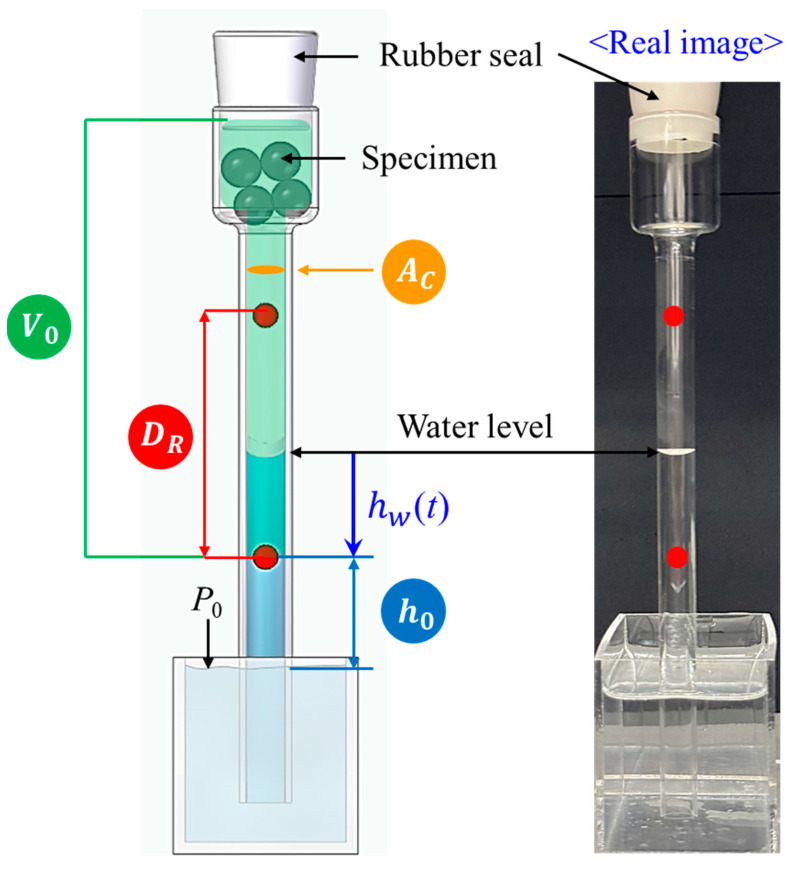
Illustrations of the parameters of the glass tube geometry used to calculate the water volume and gas amount from the water level measurements. The photo on the right shows a real image of a water-filled glass tube.

Moreover, the decrease in water level is converted into a decrease in water volume and an increase in the amount of gas, as shown on the bottom right-hand side of Figure 1b. The geometric parameters of the glass tube for the water level conversion shown in Figure 2 are introduced as follows:

- $A_{C}$ [cm^2^]: inner cross-sectional area of the thin part of the glass tube.
- $V_{0}$ [mL]: the volume of empty space between the bottom red circle marker and the rubber seal.
- $D_{R}$ [cm]: the distance between two red circle markers, designated as the water level measurement range.
- $h_{0}$ [cm]: the height from the water surface to the bottom red circle marker.

Since we limited the water level measurement range to $D_{R}$, the measured water level is denoted as $h_{w}(t)$ in Figure 2, with the bottom red circle marker representing the zero level.

The gas emitted from the specimen decreased the water volume in the glass tube as time elapsed [25,43]. As a result, the water volume and gas pressure inside the glass tube change with time. The time-varying water volume ($V_{w}(t)$) is obtained by multiplying $h_{w}(t)$ and $A_{C}$ together as

(1) $$V_{w}\left(t\right)=A_{C}h_{w}(t)$$

Considering the atmospheric pressure ($P_{0}$) in Figure 2, the time-varying gas pressure inside the glass tube ($P_{g}\left(t\right)$) is as follows:

(2) $$P_{g}\left(t\right)=P_{0}-\rho g\left[h_{w}\left(t\right)+h_{0}\right]$$

where ρ is the density of the water and g is the gravitational acceleration.

The time-varying gas volume inside the glass tube ($V_{g}\left(t\right)$) is calculated as follows:

(3) $$V_{g}\left(t\right)=V_{0}-V_{s}-V_{w}(t)$$

where $V_{s}$ is the volume of specimens.

Thus, the time-varying gas amount ($n_{g}\left(t\right)$) can be obtained via the ideal gas equation as follows:

(4) $$n_{g}(t)=P_{g}(t)V_{g}(t)/RT(t)$$

where R is the gas constant and T(t) is the absolute temperature. $n_{g}(t)$ is affected by variations in temperature and pressure in the laboratory environment. For accurate measurement of the gas amount, the variations due to changes in both temperature and pressure are compensated for according to Equation (4).

## 3. Developed Water Level Sensor

This section first describes a developed system for water level sensing with a developed program. Afterward, the sensing process of the developed sensor system is explained.

### 3.1. Water Level Sensing System

**Figure 3:**
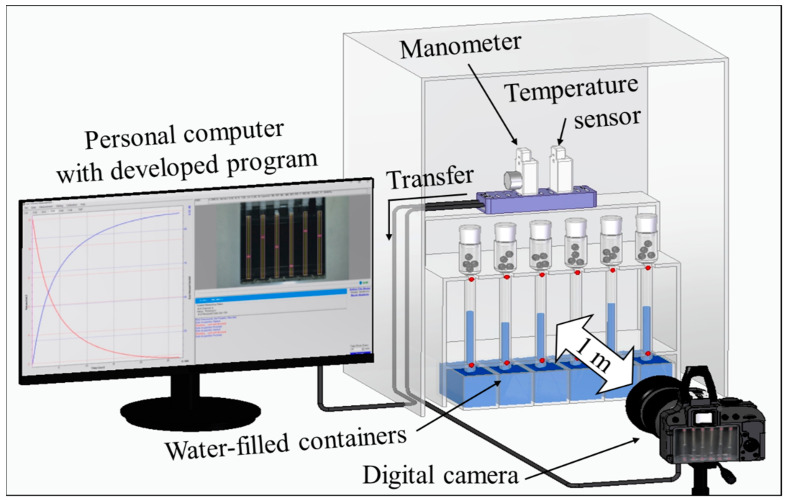
Developed water level sensing system for simultaneous measurement of six channels.

Figure 3 shows a developed water level sensing system for simultaneously measuring the water levels in six channels. The system consists of six dedicated glass tubes, a temperature sensor (UA10, DEKIST Co., Ltd., Yongin, Republic of Korea), a manometer (UA52, DEKIST Co., Ltd.), a digital camera (D800, Nikon Co., Tokyo, Japan), and a personal computer for operating the developed program. The glass tubes partially immersed in the water-filled containers were used to measure the water levels. A USB-type temperature sensor and a manometer were used to calculate the amount of outgassing from the gas-enriched specimen. The digital camera was installed 1 m away from the glass tube to minimize the distortion effect of the image. Additionally, the images captured and the measured temperature/pressure were immediately transferred to a personal computer.

### 3.2. Water Level Sensing Program

The developed program for water level sensing was designed to operate the digital camera, measure the temperature/pressure, automatically determine the water level, and display the measurement results. Visual Studio 2019 from Microsoft was used as an integrated development environment for the developed program, and the programming language C# was used.

**Figure 4:**
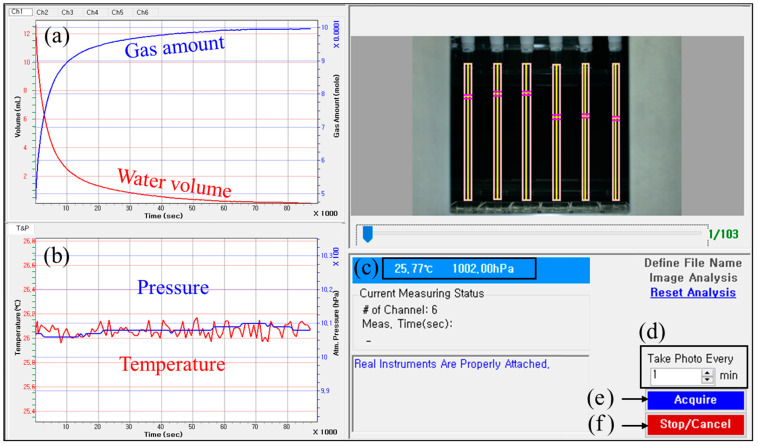
Graphical user interface (GUI) of the developed water level sensing program. (**a**) Time series for water volume (red line) and emitted gas amount (blue line) as a result of water level measurements. (**b**) Time series for the measured temperature (red line) and pressure (blue line) in the laboratory environment. (**c**) Current temperature and pressure surrounding the system. (**d**) Setting the shooting interval time of the digital camera. (**e**) Button to start measurement. (**f**) Button to stop measurement manually.

Figure 4 shows a graphical user interface (GUI) of the developed water level sensing program. Figure 4a displays the measurement results in terms of the water volume and gas amount; the red line indicates the time series of the water volume decrease computed from Equation (1), and the blue line indicates the time series of the increase in the amount of gas computed from Equation (4). Figure 4b shows the time series of the temperature (red line) and pressure (blue line) in the environment surrounding the system as measured at the same time as the results in Figure 4a. The current temperature and pressure of the system are provided in Figure 4c. The interval of the camera shooting time can be set in units of minutes up to a maximum of 60 min, as shown in Figure 4d. Figure 4e shows the button used to start the measurement. Meanwhile, the measurement time limit should be set in units of hours. The measurement is automatically terminated when the time limit is reached. The “Stop/Cancel” button in Figure 4f is used when manual termination of the measurement is required; the final measurement is performed according to the set time interval, and then the measurement is terminated.

### 3.3. Water Level Sensing Process

**Figure 5:**
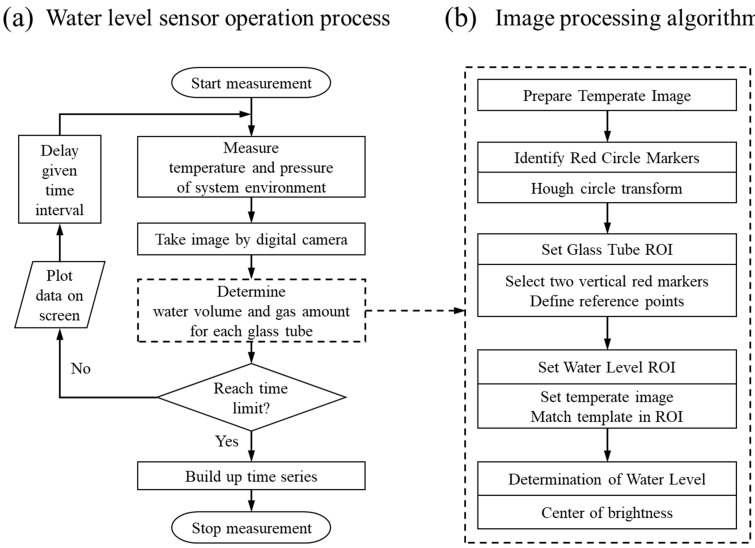
Overall water level sensing process of the developed sensor. (**a**) Flowchart of the water level measurement process using the water level sensing system and dedicated program. (**b**) Image processing algorithm for water level determination. ROI: region of interest.

Figure 5 illustrates the sensing process of the developed water level sensor. Figure 5a shows a flowchart of the water level measurement using the system in Figure 3 and the program in Figure 4. Measurement is initiated by clicking the button shown in Figure 4e. Then, the temperature/pressure surrounding the system is measured, and the water level image is simultaneously captured with a digital camera. The image is analyzed through the image processing algorithm, shown in Figure 5b and described in detail in the next section, to determine the water level. After the water level is determined, the water volume and the amount of gas in each glass tube are calculated by Equations (1) and (4), respectively. The computed water volume and the amount of gas are displayed on the program screen, and the next measurement is performed after a given time interval. This process is repeated until the time limit is reached. When the time limit is reached, the entire time series of the computed results, shown in Figure 4a, are built up. After that, the program operation is terminated.

## 4. Image Processing Algorithm

This section describes a step-by-step image processing algorithm, shown in Figure 5b. The open source “OpenCV” library was used to implement the algorithm. Each step is carried out as follows.

### 4.1. Identify Red Circle Markers

**Figure 6:**
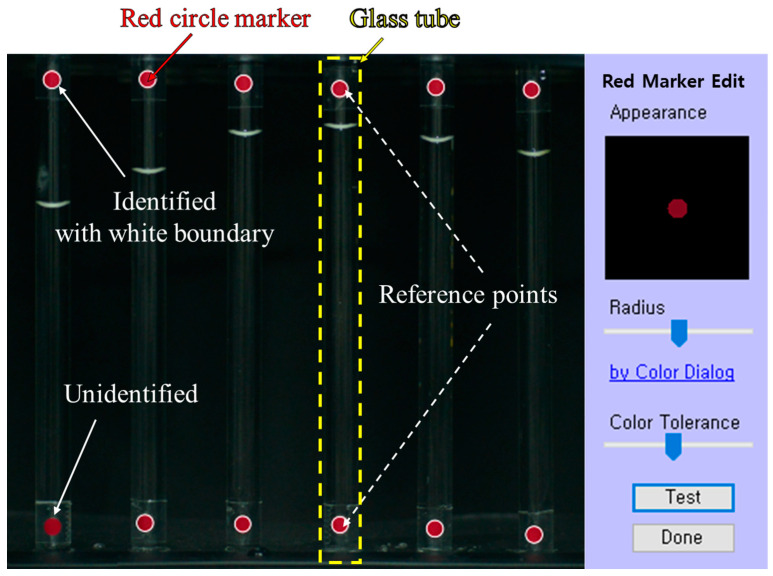
GUI for identifying the red circle markers by applying the Hough circle transform. The identified red circle markers are displayed with white circle boundaries. The yellow dotted rectangle indicates the boundary of the glass tube region. The radius and color tolerance of the identified red circle markers are adjusted via the Red Marker Edit function on the right.

The Identify Red Circle Marker step is a procedure to identify the twelve red circle markers on six glass tubes within the captured image, as shown in Figure 6. To identify the red circle markers, the Hough circle transform function is applied [46]. The identified red circle markers automatically display white circle boundaries, whereas unidentified red circle markers do not display boundaries. If not automatically identified, the user manually adjusts the radius and color tolerance for better identification via the “Red Marker Edit” function, shown on the right side of Figure 6. The radius function allows for adjustment of the identifiable size of the red circle marker. The color tolerance adjustment function was used to correct brightness changes due to lighting.

The two identified vertical red circle markers are utilized as reference points in the region where a glass tube should be located, as exemplified by the yellow dotted rectangle in Figure 6.

### 4.2. Set Glass Tube ROI

**Figure 7:**
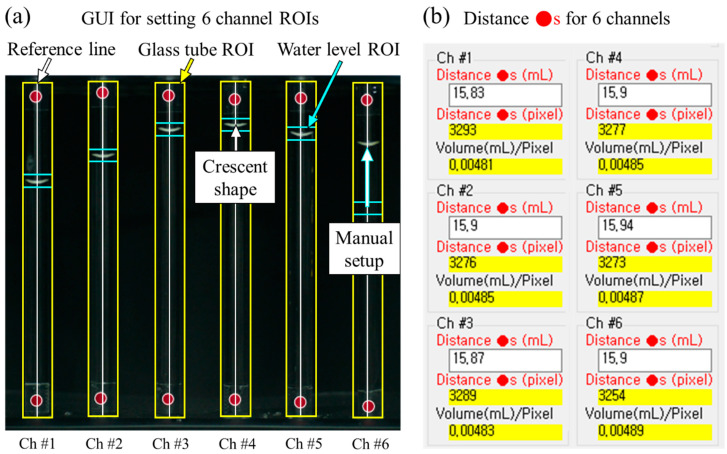
GUIs for setting the ROIs of glass tubes and water levels. (**a**) Image with determined ROIs of glass tubes and water levels. The six yellow rectangles surrounding the six white reference lines represent the glass tube ROIs. Six blue double lines with six white crescent-shaped water levels in the middle represent the water level ROIs; one on the right side is set up manually. (**b**) Volume and pixel distance between two reference points for each of the six channels. The values in “Distance ●s (mL)” are entered directly. The values of “Distance ●s (pixel)” are analyzed via image processing. Consequently, values in “Volume(mL)/Pixel” are obtained by dividing “Distance ●s (mL)” into “Distance ●s (pixel)”.

The Set Glass Tube Region of Interest (ROI) step is a procedure to recognize the six glass tubes, as displayed by the six yellow rectangles in Figure 7a. A white vertical reference line is formed by connecting the two vertical reference points. A glass tube ROI is generated surrounding the reference line. In other words, two vertical reference points and one reference line must be included within one glass tube ROI. The size of the glass tube’s ROI can be adjusted to fit the size of the glass tube used.

This step also defines the inner volume of the glass tube corresponding to one vertical pixel between the red circle markers of the six channels, as presented in Figure 7b. “Distance ●s (mL)” is the inner volume of the glass tube between the two reference points of each channel; this value is obtained by multiplying $A_{C}$ and $D_{R}$ (shown in Figure 2) and is entered directly into the program. “Distance ●s (pixel)” is the pixel distance between reference points analyzed by image processing. Therefore, water level changes can be analyzed in pixels and converted to water volume by the defined “volume (mL)/pixel”, where 0.00481 volume (mL)/pixel in Ch #1 is obtained by dividing 15.83 mL into 3293 pixels. The volume (mL)/pixel ratio in every Ch # is obtained in the same manner.

Now that the glass tube ROI is set, we can set up the water level ROI, shown by the double blue lines in Figure 7a.

### 4.3. Set Water Level ROI

The Set Water Level ROI step is a procedure to track the position of the water level inside the glass tube ROI by applying a template matching function [47]. As shown in Figure 6, the shapes of the water levels are observed as white crescent shapes in all six channels. The crescent shapes are maintained despite decreases in water level. The template matching function automatically identifies the white crescent shapes.

In Figure 7a, the blue double lines, except for Ch #6, indicate the ROIs of the identified water levels. Initially, the water level ROI must be set manually to save the crescent-shaped template in the image processing algorithm, as indicated by the white arrow of Ch #6 in Figure 7a. To sufficiently encompass the crescent shape inside the water level ROI, the height of the water level ROI is fixed to 100 pixels. After the water level ROI is set, the brightness inside the water level ROI is analyzed to precisely determine the water level.

### 4.4. Determination of Water Level

**Figure 8:**
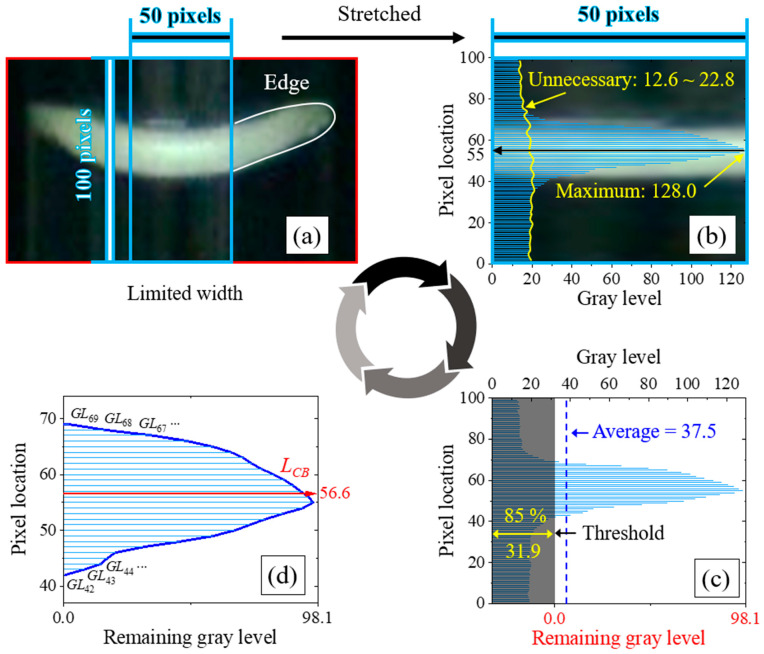
Sequence used to determine the water level by center of brightness (CB) through brightness analysis of the water level ROI. (**a**) Image with the water level ROI limited to 50 pixels wide and 100 pixels high. (**b**) A total of 100 gray levels for each vertical pixel from 0 to 99 in the limited-width image. (**c**) Setting the threshold and removing unnecessary background. (**d**) Water level determined by CM analysis of pixel location versus remaining gray levels.

Figure 8 illustrates the sequence of crescent-shaped water level determination by calculating the center of brightness (CB) for one water level ROI as follows:

(1) The blue rectangle in Figure 8a indicates that the brightness analysis region is limited to only the central 50-pixel width and 100-pixel height of the water level ROI. The edge of the crescent-shaped water level is generally steeper as the tube diameter decreases. The partial deformation of the crescent-shaped edge by the change in the glass tube diameter can affect the brightness analysis of the crescent shape. Thus, we excluded the influence of the edges by limiting the brightness analysis width of the water level ROI.
(2) The results of the brightness analysis can be expressed as gray levels ranging from 0 for black to 255 for white [48]. Figure 8b shows 100 points of gray levels corresponding to each pixel from 0 to 99 vertically in the limited-width image of 50 pixels. The magnitudes of the gray levels are expressed as a horizontal histogram, indicated by blue rods. The image behind the histogram is the limited-width image in Figure 8a stretched along the *x*-axis. According to the histogram in Figure 8b, the brightest location is presented as a maximum gray level of 128.0 at a 55-pixel location. Furthermore, unnecessary gray levels ranging from 12.6 to 22.8 were observed in the dark region of the limited-width image.
(3) The unnecessary gray levels, observed as a dark region, were removed for more accurate water level determination by setting a threshold. As shown in Figure 8c, the threshold value was defined as 31.9, which is 85% of the average (37.5) of the 100 gray levels. Thus, the gray levels below the threshold were removed. The threshold corresponding to 85% of the average was sufficient to remove the unnecessary gray levels caused by the laboratory lighting. After the removal of the threshold, the remaining gray levels are generated starting with a threshold as zero. After this process, CB analysis is performed to determine the water level using only the pixel locations versus the remaining gray levels.
(4) CB is a pixel point representing the average position of the brightness in the gray levels of the white crescent-shaped water level image. Figure 8d shows the pixel location of CB ($L_{CB}$) in the crescent shape; this location is determined by analyzing the pixel location versus the remaining gray levels. $L_{CB}$ was calculated using the following equation:
  (5) $$L_{CB}=\frac{\sum \left(i\times {GL}_{i}\right)}{\sum {GL}_{i}}$$
  where i is a pixel location and ${GL}_{i}$ is the remaining gray level corresponding to i. Thus, assuming that the water level is determined only for 100 pixels in Figure 8a, the pixel location of 56.6 ($L_{CB}$), indicated by the red arrow in Figure 8d, is determined to be the water level.

## 5. Results and Discussion

### 5.1. Measurement of the Water Level and Gas Amount

**Figure 9:**
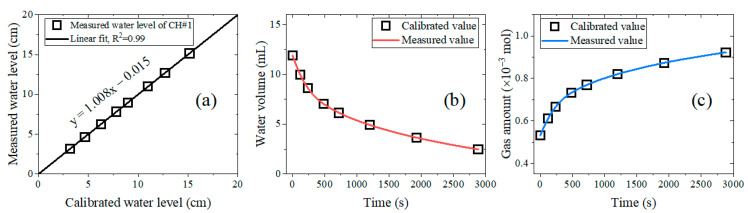
Representative water level measurement results for channel 1. (**a**) Linearity of channel 1. Linearity is expressed as a linear equation between the measured and calibrated water levels with a squared correlation coefficient (R^2^). (**b**) Measured and calibrated water volume versus elapsed time. (**c**) Measured and calibrated gas amount versus elapsed time.

The images captured by the digital camera can be distorted [49,50]. This can result in measurement errors. To investigate measurement errors that occurred due to image distortion of the developed water level sensor, we compared the measured water level with the calibrated water level using known length standards traceable to the national standard, as shown in Figure 9. The measured water level was obtained at 1-min intervals using the developed water level sensor. The calibrated water level was observed by placing a standard-length ruler immediately next to the glass tube and was obtained at the same time as the measured water level. The water levels were converted into water volume and gas amount using Equations (1) and (4), respectively. The measurement results of sensor channel 1 are representatively presented, with the channel number presented in order from left to right in Figure 7a. A glass tube with an inner diameter of 10.0 mm was used as a representative sensor. The water level was decreased by inserting the gas-enriched specimens inside the glass tube. The measurement was conducted under controlled experimental conditions, with a temperature of (25.00 ± 1.50) °C and an atmospheric pressure of (1013.00 ± 20.00) hPa.

Figure 9a shows the measured water levels versus the calibrated water levels, which are randomly selected. The linearity is defined as the slope expressed by a linear equation between the measured and calibrated water levels. The linear correlation between the measured and calibrated water levels is obtained by regression with the squared correlation coefficient, R^2^. The linearity of channel 1, which exhibited the worst linearity among all channels, was shown to be consistent within 1.0%, with R^2^ = 0.99. This indicates that the water level determination performance of the image processing algorithm described in Section 4 exhibited good linearity, exceeding 99%. Meanwhile, Figure 9b,c show the time-varying decrease in water volume and the increase in gas amount. The calibrated water volume and gas amount were calculated from the calibrated water levels shown in Figure 9a. The measured values are computed by the program. Good agreement between the measured and calibrated values confirmed that the sensing algorithm shown in Figure 5 operates well and without errors in building the time series results.

**Figure 10:**
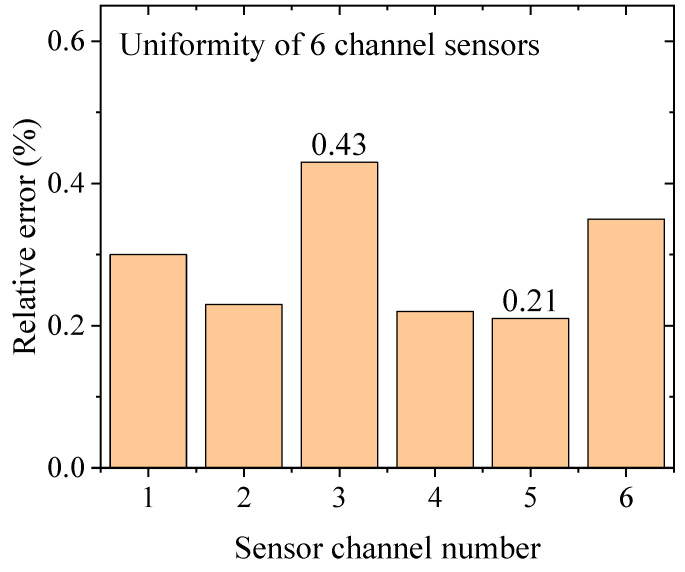
Uniformity of six channels expressed as the relative error between the measured and calibrated water levels at the maximum measurement height.

Figure 10 shows the relative error between the measured water level and the calibrated water level at the maximum measurement height of each channel. Uniformity refers to the degree of similarity between these relative errors in the six channels. The relative error was almost uniform within 0.5% for all channels, ranging from a minimum of 0.21% in channel 5 to a maximum of 0.43% in channel 3. Since the distortion of the captured image is most evident at the edges [49,50], the relative error due to the distortion should be largest in channels 1 and 6. However, the fact that the highest relative error was found in channel 3 implies that the error occurred for another reason; it may have occurred when the distances of the vertical red circle markers shown in Figure 2 were measured.

### 5.2. Performance Test for the Developed Water Level Sensor

We tested the various performance levels of the developed water level sensor. The performance tests examined the sensor’s accuracy, sensitivity, resolution, stability, measurement range, response time, and cost. A representative performance evaluation of a glass tube with an inner diameter of 10 mm is employed and summarized in Table 1 with the performance results of other types of commercial sensors. The performances of the commercial sensors, including ultrasonic [51] and capacitive [52] sensors, were obtained from the specifications provided by the manufacturers and refer to performance under specific conditions such as room temperature and atmospheric pressure.

The accuracy is defined as the degree to which a measured value agrees with the actual value [53]. Thus, we expressed the accuracy as the relative error of the water level measured by the sensor corresponding to the calibrated water level. The maximum vertical relative error in Figure 9a is 0.8%, and the maximum horizontal relative error in Figure 10 is 0.4%. According to the propagation of the error formula [54,55], the accuracy is estimated to be 0.9%.

The sensitivity is defined as the change in the water level with respect to the change in the pixel distance [56]. “Volume (mL)/Pixel” in Figure 7b represents the volumetric sensitivity of the six channel sensors. The sensor’s sensitivity was determined to be 0.005 mL/pixel, which is the maximum volumetric sensitivity of the six channels. For comparison with other commercial sensors in units of length, the maximum volumetric sensitivity is divided by the cross-sectional area of the glass tube. Thus, the sensitivity obtained was 0.06 mm/pixel, as shown in Table 1.

A sensor with high sensitivity implies better resolution. The resolution is indicative of the minimum measurable value, which corresponds to the length calculated from a single pixel measurement of the digital camera image [43]. The image processing algorithm that we developed calculates fine water level changes in a unit of 0.1 pixel using the water level determination sequence shown in Figure 8. The water level determination is based on the image brightness measurement. Since the brightness measurement of the image is performed in a unit of 1 pixel, the water level corresponding to 1 pixel is determined as the resolution. Thus, the resolution is 0.06 mm.

The stability of the sensor system is defined as the standard deviation obtained from measurements over 24 h after the outgassing is terminated, which corresponds to 0.2–0.3% of the water level. Since the stability was consistent during extended measurements of up to 168 h in a laboratory environment, we presented the 24-h measurement as a representative indicator of short-term stability. Meanwhile, the measurement range represents the maximum allowable length of the glass tube that can be captured in one image. The maximum number of pixels that can be contained in one image is 4742. Thus, the maximum measurement range of the evaluated sensor is 0.3 m, which can be extended. The measurement range of 0.3 m is suitable for laboratory-scale applications such as the measurement of gas emissions from enriched specimens. However, the range of 0.3 m was unsuitable for applications where significant gas flow or outgassing occur. Therefore, as the scale of practical applications increases, the size of the glass tube needs to be enlarged. The response time is defined as the time required for a digital camera to capture an image and subsequently display the measurement results. Thus, the process instantly responds after no more than 1.0 s.

The total cost of the developed laboratory-scale water level sensor via the image processing technique is USD 80.00, including the cost of manufacturing six glass tubes (USD 7.30) and the program purchase cost of USD 72.70. Therefore, the total cost of the proposed water level measurement system, which includes sensors and a sensing program, is much lower than the cost of commercially available ultrasonic water level sensors (USD 480) and capacitive water level sensors (USD 110).

As shown by a performance comparison, the developed sensor showed superior performance, with a resolution of 0.06 mm and a low cost of USD 80, compared with the ultrasonic-type and capacitive-type sensors, as listed in Table 1.

## 6. Conclusions

A water level sensor system and image processing algorithm were developed to simultaneously detect six white crescent-shaped water levels via images captured by a digital camera. The water level sensor exhibits the following particular characteristics: an accuracy of 0.9%; a water height detection limit of 0.06 mm; a stability of 0.3%; an upper maximum measurement range of 0.3 m; fast response times, within a second; and a low cost of USD 80. In conclusion, the features of the developed sensor technique are summarized below.

(1) An effective high-resolution and low-cost technique for measuring water level and gas concentration;
(2) An automatic measurable technique involving the application of a dedicated water level sensing program;
(3) A sophisticated image processing algorithm of the water level by calculating the center of brightness of the white crescent-shaped water level;
(4) A visible technique to immediately track changes in the water level and gas emission.

The developed image-based sensor was designed to measure a decrease in water level due to gradual outgassing of a water-insoluble, gas-enriched specimen. The water-insoluble gases include hydrogen, helium, argon, nitrogen, and oxygen. Our sensor can be utilized to measure the amount of all emitted water-insoluble gases as well as the water level. In particular, measurements of the permeation and leakage of hydrogen are necessary in plastic pipes and sealing O-rings when high-pressure gaseous hydrogen is stored in the hydrogen infrastructure. To solve these issues, the developed sensor is applied to effectively quantify permeation properties, such as the diffusivity, solubility, and permeability, of high-pressure hydrogen in polymer materials using high-resolution and low-cost image processing techniques. In addition, we are attempting to apply continuous gas flow measurements for incidents such as hydrogen leakage to plastic hydrogen pipelines.

## Figures and Tables

**Table 1:** Performance comparison of several water level sensors.

Performance Item	Developed Water Level Sensor	Ultrasonic Water Level Sensor [51]	Capacitive Water Level Sensor [52]
Accuracy	0.9%	0.5%	0.5%
Sensitivity	0.06 mm/pixel	not specified	not specified
Resolution	0.06 mm	1 mm	not specified
Stability	0.3%	not specified	0.2%
Measurement range	0.3 m	5.0 m	10.0 m
Response time	1.0 s	0.5 s	0.5 s
Cost	USD 80	USD 480	USD 110

## Data Availability

The data used to support the findings of this study are available from the corresponding author upon request.

## References

[1] Joshi P.C., Chopade N., Chhibber B. Liquid level sensing and control using inductive pressure sensor. Proceedings of 2017 the International Conference on Computing, Communication, Control and Automation (ICCUBEA).

[2] Liu S., Tian J., Liu N., Xia J., Lu P. (2016). Temperature insensitive liquid level sensor based on antiresonant reflecting guidance in silica tube. J. Light. Technol..

[3] Liu X., Bamberg S., Bamberg E. (2011). Increasing the accuracy of level-based volume detection of medical liquids in test tubes by including the optical effect of the meniscus. Measurement.

[4] Meera C., Sunny S., Singh R., Sairam P.S., Kumar R., Emannuel J. Automated precise liquid transferring system. Proceedings of the 2014 IEEE 6th India International Conference on Power Electronics (IICPE).

[5] Loizou K., Koutroulis E. (2016). Water level sensing: State of the art review and performance evaluation of a low-cost measurement system. Measurement.

[6] Antunes P., Dias J., Paixão T., Mesquita E., Varum H., André P. (2015). Liquid level gauge based in plastic optical fiber. Measurement.

[7] Marques C.A., Peng G.-D., Webb D.J. (2015). Highly sensitive liquid level monitoring system utilizing polymer fiber Bragg gratings. Opt. Express.

[8] Alsdorf D.E., Melack J.M., Dunne T., Mertes L.A., Hess L.L., Smith L.C. (2000). Interferometric radar measurements of water level changes on the Amazon flood plain. Nature.

[9] Kim S.-W., Choi H.-S., Park D.-U., Baek E.-R., Kim J.-M. (2018). Water level response measurement in a steel cylindrical liquid storage tank using image filter processing under seismic excitation. Mech. Syst. Signal Process..

[10] Nair B.B., Rao S. (2016). Flood water depth estimation—A survey. Proceedings of the 2016 IEEE International Conference on Computational Intelligence and Computing Research (ICCIC).

[11] Ridolfi E., Manciola P. (2018). Water level measurements from drones: A pilot case study at a dam site. Water.

[12] Liu M., Wang C., Huang W., Wang X., Li S., Lu P., Liu X., Jiang E. (2024). Research on water level measurement technology based on the residual length ratio of image characters. Signal Image Video Process..

[13] Circone S., Kirby S., Pinkston J., Stern L. (2001). Measurement of gas yields and flow rates using a custom flowmeter. Rev. Sci. Instrum..

[14] Pavani G.J., Pavani S.A., Ferreira C.A. (2021). Gas permeameter in polymer nanocomposite plates: Construction and validation. Iran. Polym. J..

[15] Jung J.K., Kim I.G., Kim K.T., Ryu K.S., Chung K.S. (2021). Evaluation techniques of hydrogen permeation in sealing rubber materials. Polym. Test..

[16] Jung J.K., Kim I.G., Jeon S.K., Kim K.-T., Baek U.B., Nahm S.H. (2021). Volumetric analysis technique for analyzing the transport properties of hydrogen gas in cylindrical-shaped rubbery polymers. Polym. Test..

[17] Vasiliev A., Pisliakov A., Sokolov A., Polovko O., Samotaev N., Kujawski W., Rozicka A., Guarnieri V., Lorencelli L. (2014). Gas sensor system for the determination of methane in water. Procedia Eng..

[18] Luo Y., Hu L., Ochs F., Tosatto A., Xu G., Tian Z., Dahash A., Yu J., Yuan G., Chen Y. (2023). Semi-analytical modeling of large-scale water tank for seasonal thermal storage applications. Energy Build..

[19] Sahoo A.K., Udgata S.K. (2019). A novel ANN-based adaptive ultrasonic measurement system for accurate water level monitoring. IEEE Trans. Instrum. Meas..

[20] Zakaria Z., Idroas M., Samsuri A., Adam A.A. (2017). Ultrasonic instrumentation system for Liquefied Petroleum Gas level monitoring. J. Nat. Gas Sci. Eng..

[21] Stateczny A. Radar water level sensors for full implementation of the river information services of border and lower section of the Oder in Poland. Proceedings of the 2016 17th International Radar Symposium (IRS).

[22] Jung J., Kim G., Gim G., Park C., Lee J. (2022). Determination of gas permeation properties in polymer using capacitive electrode sensors. Sensors.

[23] Ohira S.-I., Goto K., Toda K., Dasgupta P.K. (2012). A capacitance sensor for water: Trace moisture measurement in gases and organic solvents. Anal. Chem..

[24] Loizou K., Koutroulis E., Zalikas D., Liontas G. A low-cost capacitive sensor for water level monitoring in large-scale storage tanks. Proceedings of the 2015 IEEE International Conference on Industrial Technology (ICIT).

[25] Jung J.K., Lee J.H. (2024). High-performance hydrogen gas sensor system based on transparent coaxial cylinder capacitive electrodes and a volumetric analysis technique. Sci. Rep..

[26] Zhang Z., Zhou Y., Liu H., Gao H. (2019). In-situ water level measurement using NIR-imaging video camera. Flow Meas. Instrum..

[27] Hou Y., Li Q., Zhang C., Lu G., Ye Z., Chen Y., Wang L., Cao D. (2021). The State-of-the-Art Review on Applications of Intrusive Sensing, Image Processing Techniques, and Machine Learning Methods in Pavement Monitoring and Analysis. Engineering.

[28] Liu J.G., Mason P.J. (2024). Image Processing and GIS for Remote Sensing: Techniques and Applications.

[29] Lan H., Song Z., Bao H., Ma Y., Yan C., Liu S., Wang J. (2024). Shear strength parameters identification of loess interface based on borehole micro static cone penetration system. Geoenviron. Disasters.

[30] Mennel L., Symonowicz J., Wachter S., Polyushkin D.K., Molina-Mendoza A.J., Mueller T. (2020). Ultrafast machine vision with 2D material neural network image sensors. Nature.

[31] Lan H., Liu X., Li L., Li Q., Tian N., Peng J. (2022). Remote sensing precursors analysis for giant landslides. Remote Sens..

[32] Zhang Z., Zhu L. (2023). A review on unmanned aerial vehicle remote sensing: Platforms, sensors, data processing methods, and applications. Drones.

[33] Alakarhu J. (2007). Image sensors and image quality in mobile phones. International Image Sensor Workshop.

[34] Suzuki T. Challenges of image-sensor development. Proceedings of the 2010 IEEE International Solid-State Circuits Conference-(ISSCC).

[35] Qiao G., Yang M., Wang H. (2022). A water level measurement approach based on YOLOv5s. Sensors.

[36] Wang X., Li Z., Zhang Y., An G. (2023). Water level recognition based on deep learning and character interpolation strategy for stained water gauge. River.

[37] Gilmore T.E., Birgand F., Chapman K.W. (2013). Source and magnitude of error in an inexpensive image-based water level measurement system. J. Hydrol..

[38] Yun H.S., Jeon S.K., Lee Y.-K., Park J.S., Nahm S.H. (2024). Effect of precharging methods on the hydrogen embrittlement of 304 stainless steel. Int. J. Hydrogen Energy.

[39] Lee J.-H., Kim Y.-W., Jung J.-K. (2023). Investigation of the Gas Permeation Properties Using the Volumetric Analysis Technique for Polyethylene Materials Enriched with Pure Gases under High Pressure: H_2_, He, N_2_, O_2_ and Ar. Polymers.

[40] Lee J.H., Kim Y.W., Chung N.K., Kang H.M., Moon W.J., Choi M.C., Jung J.K. (2024). Multiphase modeling of pressure-dependent hydrogen diffusivity in fractal porous structures of acrylonitrile butadiene rubber-carbon black composites with different fillers. Polymer.

[41] Jung J.K., Kim I.G., Chung K.S., Baek U.B. (2021). Gas chromatography techniques to evaluate the hydrogen permeation characteristics in rubber: Ethylene propylene diene monomer. Sci. Rep..

[42] Jung J.K., Lee J.H., Park J.Y., Jeon S.K. (2024). Modeling of the Time-Dependent H2 Emission and Equilibrium Time in H2-Enriched Polymers with Cylindrical, Spherical and Sheet Shapes and Comparisons with Experimental Investigations. Polymers.

[43] Jung J.K., Kim K.-T., Lee J.H., Baek U.B. (2023). Effective and low-cost gas sensor based on a light intensity analysis of a webcam image: Gas enriched polymers under high pressure. Sens. Actuators B Phys..

[44] Jung J.K. (2024). Review of Developed Methods for Measuring Gas Uptake and Diffusivity in Polymers Enriched by Pure Gas under High Pressure. Polymers.

[45] Thomas A.M., Cross J.L. (1972). Micrometer U-tube manometers for medium-vacuum measurements. NBS Spec. Publ..

[46] Mukhopadhyay P., Chaudhuri B.B. (2015). A survey of Hough Transform. Pattern Recognit..

[47] Brunelli R. (2009). Template Matching Techniques in Computer Vision: Theory and Practice.

[48] Bovik A.C. (2009). Basic gray level image processing. The Essential Guide to Image Processing.

[49] Creutin J., Muste M., Bradley A., Kim S., Kruger A. (2003). River gauging using PIV techniques: A proof of concept experiment on the Iowa River. J. Hydrol..

[50] Muste M., Fujita I., Hauet A. (2008). Large-scale particle image velocimetry for measurements in riverine environments. Water Resour. Res..

[51] ATO Ultrasonic Water Level Sensor (ATO-LEVS-ZP). https://www.ato.com/ultrasonic-level-sensor-40m.

[52] Apure KS-SMY1 Hydrostatic Water Level Sensor. https://apureinstrument.com/level-measurement/hydrostatic-level-sensor/ks-smy1-hydrostatic-water-level-sensor/.

[53] Makridakis S. (1993). Accuracy measures: Theoretical and practical concerns. Int. J. Forecast..

[54] Ku H.H. (1966). Notes on the use of propagation of error formulas. J. Res. Natl. Bur. Stand..

[55] Trisna B.A., Park S., Lee J. (2024). Significant impact of the covid-19 pandemic on methane emissions evaluated by comprehensive statistical analysis of satellite data. Sci. Rep..

[56] Saltelli A. (2002). Sensitivity analysis for importance assessment. Risk Anal..

